# Differentiating between Affine and Perspective-Based Models for the Geometry of Visual Space Based on Judgments of the Interior Angles of Squares

**DOI:** 10.3390/vision2020022

**Published:** 2018-06-02

**Authors:** Mark Wagner, Gary Hatfield, Kelly Cassese, Alexis N. Makwinski

**Affiliations:** 1 Department of Psychology, Wagner College, Staten Island, NY 10301, USA; 2 Department of Philosophy, University of Pennsylvania, Philadelphia, PA 19104, USA

**Keywords:** visual space, affine transformations, perspective transformations, angle judgments, visual geometry, space perception

## Abstract

This paper attempts to differentiate between two models of visual space. One model suggests that visual space is a simple affine transformation of physical space. The other proposes that it is a transformation of physical space via the laws of perspective. The present paper reports two experiments in which participants are asked to judge the size of the interior angles of squares at five different distances from the participant. The perspective-based model predicts that the angles within each square on the side nearest to the participant should seem smaller than those on the far side. The simple affine model under our conditions predicts that the perceived size of the angles of each square should remain 90°. Results of both experiments were most consistent with the perspective-based model. The angles of each square on the near side were estimated to be significantly smaller than the angles on the far side for all five squares in both experiments. In addition, the sum of the estimated size of the four angles of each square declined with increasing distance from the participant to the square and was less than 360° for all but the nearest square.

## 1. Introduction

Space perception is one of the oldest and most deeply investigated areas of perceptual psychology. The vast majority of empirical research on space perception is unidimensional in nature. For example, one set of studies might look at size perception of only frontally oriented targets, another looks at only flat targets, oriented in-depth, and yet another only at egocentric distance judgments. In addition, for over a century researchers have examined the effects of judgment method, instructions, and the meaning given to concepts like size and size constancy. (See [1,2] for meta-analyses of work concerning the direct estimation of spatial metrics, size-constancy research, and the role of instructions.) Only a relatively small subset of space perception research has attempted to describe the geometry of visual space as a whole.

Researchers have suggested a number of geometries for visual space ([3] provides a review). For example, Gibson’s [4,5] doctrine of realism implies that visual space should be strictly Euclidean because our perceptions ought to match Euclidean physical reality. Thomas Reid [6] suggested that visual space follows a spherical geometry; a result supported by some recent philosophers ([7] (Chapter 3), provides a review and interpretation). Hoffman and Dodwell [8,9] believe it displays the properties of a Lie transformation group. Drösler [10,11,12] thought it was a Cayley-Klein geometry. 

Most famously, Luneburg [13,14,15] applied a hyperbolic geometry to visual space in order to explain Hillebrand [16] and Blumenfeld’s [17] parallel alley experiments, which found that in order to produce apparently parallel lines or walls, subjects adjusted the lines or walls so that they diverged as they receded from the observer. After Luneburg’s death in 1949, Blank [18,19,20] explicitly laid out the assumptions underlying Luneburg’s theory and marshaled evidence in their support. He believed that visual space satisfied the axioms of a geometry of constant curvature and that this curvature is negative, consistent with the hyperbolic formulation. Indow [21,22,23,24,25] subsequently tested Luneburg’s theory under varying cue conditions, stimulus orientations, and settings which generally supported the idea that visual space had a negative curvature, as Luneburg had proposed. More recently, Heller [26,27] and Aczél et al. [28] have introduced additional refinements to the theory.

Some authors question the existence of any well-defined visual geometry, citing demonstrations that spatial judgments can be affected by changes in context and the presence of reference frames [29,30,31,32]. However, Wagner [3] suggests that these recent results, rather than proving that visual space doesn’t exist, simply offer new instances in the long sequence of studies showing that spatial judgments and visual space itself may change with context. Therefore, statements about the geometry of visual space are always in reference to a specific set of instructions and stimulus conditions.

Past work on the geometry of visual space follows one of two research strategies. Luneburg, Blank, and others attempt to describe the intrinsic structure of visual space by looking at how the properties of perceived space relate to each other without reference to the physical layout of stimuli. Others take an extrinsic approach in which visual space is defined in terms of a transformation of physical space. The present work follows the extrinsic, transformational strategy.

This study concerns two competing accounts of the transformation of physical space into visual space: that visual space reflects a simple affine transformed Euclidean space (Wagner and colleague [3,33,34]), or that it is shaped by the laws of perspective (Erkelens [35,36,37,38,39]; Gilinsky [40]; Hatfield [41,42,43]). It seeks to distinguish empirically between these two models through two studies concerning judgments of the interior angles of squares lying on the ground.

## 2. Visual Space as an Affine Transformation

One recent model for visual space treats it as the product of a simple affine transformation of physical space. The in-depth dimension of visual space is perceptually compressed (or occasionally expanded) compared to the frontal dimension of visual space (and compared with physical space). However, after the transformation, visual space is still thought to be essentially Euclidean; parallel lines remain parallel and collinearity is preserved.

For example, Wagner [33,44] pounded stakes randomly in a large, flat, grassy field, and asked observers to judge distances between pairs of stakes and angles formed by stake triplets using four different psychophysical methods. He found that stimulus orientation strongly affected judgments in the same way for all judgment methods. For stimuli the same physical distance apart, those between-stake distances receding away from the observer in depth were seen to be half as large on average as those oriented frontally with respect to the observer. Angle judgments showed a similar pattern. For angles of the same physical size, those whose open ends faced either toward or away from the observer appeared to expand perceptually, while those whose open ends faced to the right or left (the observer looks across the legs of the angle) appeared to contract perceptually. Accordingly, the judged angle is larger than the physical angle for angles that face toward or away from the observer, while the judged angle is smaller than the physical angle for angles facing off to the side.

Wagner [33,44] applied 12 candidate metrics to describe these data and found that two of them fit judgments much better than the others. The first was a simple Affine Contraction model. In this model, the observer is placed at the origin of a Euclidean plane with the x-axis corresponding to the left–right frontal dimension and the y-axis corresponds to the observer’s in-depth dimension. According to the model, the frontal dimension is accurately perceived, while the in-depth dimension is perceptually compressed. After the transformation, the space is still Euclidean. This leads to the following formula to describe the relationship between perceived distance, *s′*, and the physical coordinates of the two end points (*x*_1_, *y*_1_) and (*x*_2_, *y*_2_):

(1) $$s^{\prime }=\sqrt{{\left(x_{1}-x_{2}\right)}^{2}+{\left(c\left(y_{1}-y_{2}\right)\right)}^{2}}$$

where *c* reflects the degree of compression of the in-depth dimension of visual space. Wagner found that all judgment methods displayed very similar amounts of compression, and on average *c* = 0.45. In other words, a physical stimulus oriented in-depth seemed to be less than half as large as the same physical stimulus oriented frontally. Using a formula from Riemannian geometry, the same model was applied to the angle data, and it yielded a similar degree of compression across all methods that corresponded closely to the value of *c* obtained with distance judgments. For angle judgments, on average *c* = 0.48.

Wagner and Feldman [45] extended this work to three dimensions, under both light and dark viewing conditions. (See [3] for details.) Under full-cue conditions, the compression parameter averaged *c* = 0.52 for distance judgments and *c* = 0.62 for angle judgments. The degree of compression was even more extreme under reduced cue conditions with *c* = 0.35 for distance judgments and *c* = 0.32 for angle judgments.

Wagner discussed a second model, called the Vector Contraction model. In this model, distances in visual space can be decomposed into frontal and in-depth components and the in-depth component is compressed in visual space. Unlike the simple Affine Contraction model, parallels are not preserved. The Vector Contraction model produced similar degrees of compression and fit the data slightly better than the Affine Contraction model. 

In subsequent years, numerous papers have suggested that visual space displays an affine transformed structure [3,46,47,48,49,50,51,52,53,54,55,56,57,58,59,60,61,62,63]. However, these studies show that visual space is not as simple as the affine model suggests since the amount of compression in the in-depth dimension of visual space varies considerably from one study to another. The critical variable determining the degree of compression in the in-depth dimension appears to be distance. Wagner and Gambino [34] performed a meta-analysis of past studies that examined this phenomenon and concluded that, for stimuli nearer than 1 m away, the in-depth dimension of visual space appears to expand relative to the frontal dimension (*c* > 1); however, for stimuli more than 1 m away from the observer, the in-depth dimension of visual space is compressed relative to the frontal dimension (*c* < 1). The compression parameter quickly declines as distance to the stimulus increases, but the rate of change slows beyond 7 m from the observer, reaching an apparent asymptote at about *c* = 0.5 for more distant stimuli. In addition, the compression parameter for visual space is smaller under monocular and reduced-cue conditions. Wagner and Gambino also confirmed this pattern directly through an experiment that systematically measured the size of the compression parameter as a function of distance to the stimulus.

Wagner and Gambino suggested that the pattern of compression in the in-depth dimension as a function of distance is similar to the ratio of in-depth to frontal visual angles of stimuli, but is not as extreme as this ratio, implying that observers are incapable of fully ignoring size information provided by cues to depth.

If the compression parameter varies as a function of distance, Equation (1) no longer corresponds to an affine-transformed space since length proportions of line intervals and parallelism of physical space will generally no longer be preserved after the transformation. However, since the actual function relating compression to distance is not well defined, it is not possible to make concrete predictions about the exact structure of visual space based upon this more complicated model. On the other hand, a simple affine model (with a fixed compression parameter) makes clear experimental predictions in the current experimental context. Thus, to simplify the comparison with perspective-based models, the current work contrasts the predictions of the simple affine model with those of a perspective-based approach. (Having said this, although we are focusing on the simple affine model in the paper, we believe the specific experimental layout used in the present experiment should produce the same experimental predictions for the model with variable compression as the simple affine model.)

The present study looks at judgments of the interior angles of squares. A simple affine model makes a number of predictions about these judgments. First of all, most affine models assume that visual space is Euclidean after the transformation; parallels are preserved, as is collinearity. The sum of the perceived angles of a square should be 2π or 360°. Secondly, if the sides of a physical square were parallel to the frontal (x-axis) and in-depth (y-axis) axes of visual space, then the affine transformation should simply transform the perceived square into a rectangle. The ratio of the in-depth to frontal dimensions of the perceived rectangle would depend on the distance to the object from the observer, but at all distances the perceived object should look rectangular. For this reason, in an affine transformed space, each of the interior angles of the rectangle should continue to be perceived to be 90°. (Of course, this prediction is only possible for squares with sides oriented parallel to the observer’s frontal and medial planes. If the squares were oriented from any other perspective, the affine model would predict changes in apparent angle. That is why we orient the squares as we do.) In addition, the affine model predicts that, for a given rectangle, the angles nearer to the observer should seem perceptually equal to the angles that are more distant from the observer.

As previously mentioned, Wagner [33,44] and Wagner and Feldman [45] had two models for visual space that worked, the Affine Contraction model and the Vector Contraction model. Wagner could not differentiate between these models with his data. Since the two models seem so similar, the Vector Contraction model has received little attention. However, the two models predict different perceptual responses to the aforementioned square. The Vector Contraction model predicts that the near angles of the square would be perceptually compressed, that is, be less than 90°, and that the far angles of the square would expand, that is, be greater than 90°. The four interior angles of the quadrilateral would still sum up to 360° (under the assumption of a Euclidean Vector transformation).

Since the Vector Contraction model makes predictions similar to perspective-based models in the current experimental context, the present work focuses on the clear contrast between the predictions of the simple affine model and those of the perspective-based approach.

## 3. Linear Perspective-Based Models for Visual Space

Recently, the philosopher Hatfield [41,42,43] has advanced a theory for visual space deriving from his careful phenomenological observations. Hatfield began from a simple phenomenological observation: straight stretches of railway tracks, sidewalks, streets, and hallways appear to converge with distance. This commonplace observation had not been well described by some of the major visual theorists. In different ways, both Gibson [4] and Rock ([64] (pp. 562–563); [65] (pp. 339–349); [66] (pp. 254–265)) assimilated this phenomenon to the perspective projection of a three-dimensional scene onto a two-dimensional plane, that is, to ordinary linear perspective.

A geometrical structure created by ordinary linear perspective does not, however, fit the phenomenal experience of looking down the railway tracks, sidewalk, street, or hallway. For although the tracks (and the edges of the sidewalk, etc.) appear to converge, they do not appear to be located in a two-dimensional plane that is perpindicular to the line of sight (and located somewhere in front of the observer), as specified by ordinary linear perspective. Rather, the tracks converge as they run away in depth. The narrower and narrower tracks are phenomenally experienced as being farther and farther away. Thus, a geometrical structure is needed that both converges and includes phenomenal depth, or the third dimension.

Hatfield cast about for existing models that would account for this fact. He was not attracted to non-Euclidean models, as these often assume, as did Luneburg, that the non-Euclidean physical space is of constant curvature, which may be a desirable assumption for descriptions of physical space but need not and may not obtain for visual space (see also [3] (pp. 31–36)). He was not attracted to affine theories. Such models have the disadvantage of not preserving visual direction. That is, as space is compressed, visual directions from the observer should become more widely separated (subtend a wider angle in a horizontal plane at eye-level) than the corresponding physical directions. But visual direction is perceived with great accuracy. Although it can be slightly modified by eye occlusion, eye position, and ocular dominance, it is generally consistent with Hering’s law of visual direction [67].

Hatfield also noted that, phenomenally, the floor in the hallway and the sidewalk in front of the observer appear to rise with distance. (This had been noted by the philosopher Slomann [68] and more recently by the psychologists Ooi and He [69].) Hatfield sought a geometrical stucture that would capture the convergence of railway tracks in depth and the rising of the floor, while preserving visual direction.

**Figure 1:**
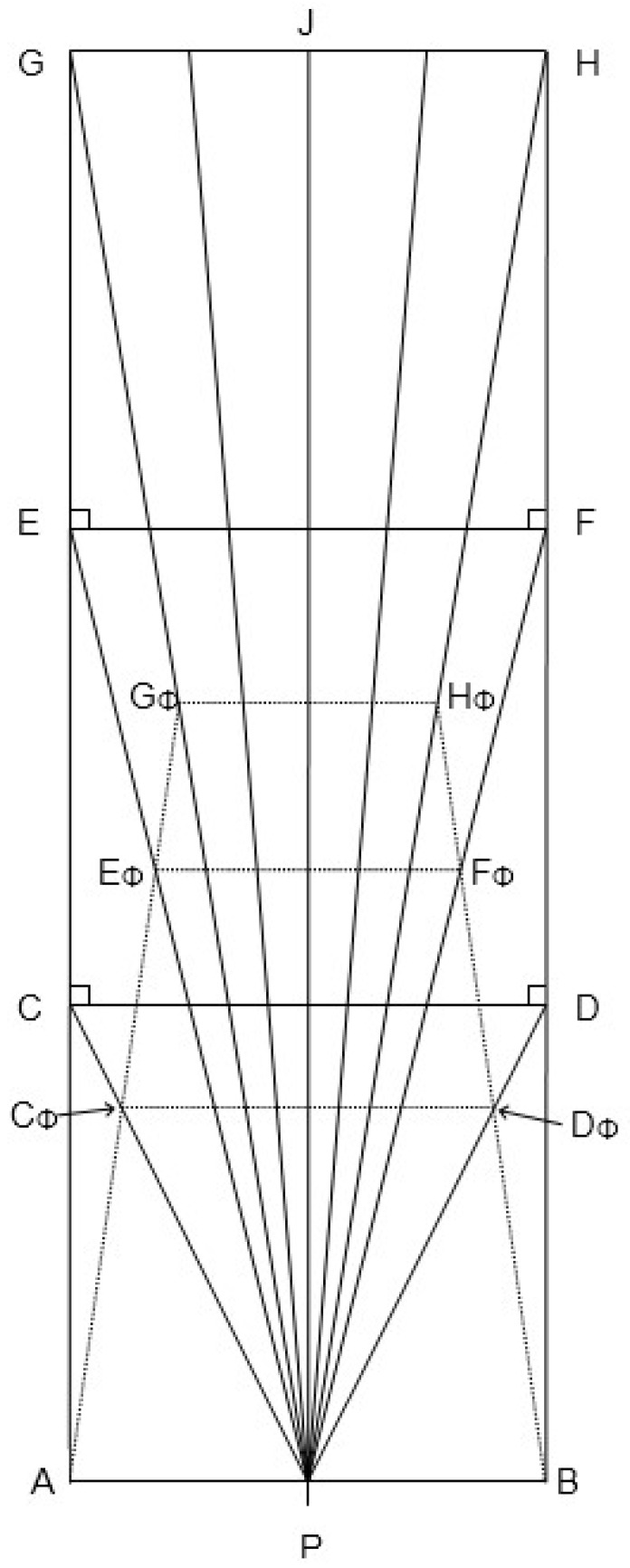
Diagram illustrating the spatial contraction proposed in Hatfield’s Perspective-Based model.

Hatfield [41] devised a model that is related to linear perspective. Visual space is contracted in relation to physical space along the lines of sight, as illustrated in Figure 1. The diagram represents a horizontal plane that bisects the eyes of an upright observer located at P. The outer solid lines represent the physical walls in a rectangular hallway. The lines from P to points C–J are lines of sight. The dotted lines AC_Φ_–BH_Φ_ represent the location of the walls as experienced in visual space, and the dotted lines C_Φ_–D_Φ_ and so on represent perpindicular cross-sections. The walls appear to converge in depth (and the floor to raise, if we take the diagram to reprent a saggital or median plane). Visual direction is preserved. The phenomenal space is contracted with respect to physical space. This contraction is greater in the in-depth dimension than it is for planes situated perpendicular to the line of sight—that is, the phenomenal counterpart to segment EG is more compressed than is the counterpart to segment GH. The model is related to linear perspective in that physical locations are projected along lines of sight. It is a three-dimensional to (contracted) three-dimensional projection. A two-dimensional perspective projection is obtained by considering the scene as projected onto a plane that cuts the lines of sight EP, FP, etc. perpendicularly with respect to the central line of sight, PJ. In the perspective projection onto the plane, the sides of the hallway would converge, but this convergence is contained in the plane of projection and so does not recede in depth.

Considering these observations suggests that for moderate and far distances (more than about 1 m away from the observer), visual experience does not exhibit phenomenal size constancy. The word “phenomenal” in *phenomenal size constancy* means the sizes that objects appear to have, rather than the sizes that they are judged to have when observers are asked to estimate objective physical size. As Boring [70] noted long ago, our experience of objects at a distance is paradoxical. On the one hand, in appearance, objects at a distance look smaller than the same objects near at hand. On the other hand, we can often tell from how they look what their objective sizes are (to some degree of accuracy). Railway tracks appear phenomenally to converge, but we also believe that they are parallel. The present investigation focuses on spatial appearances, rather than on judgments taken under instructions to report the objective physical properties of things.

The situation in which an observer would experience full phenomenal constancy is also reprsented in Figure 1. If an observer experienced full phenomenal size constancy, the walls of the hallway would appear where they are, physically. That is, phenomenal segment E_Φ_G_Φ_ would appear at the location where physical segment EG is found. Accordingly, there would be no phenomenal contraction of the hallway amd its sides would not seem to converge (and similarly for the railway tracks). Hatfield observed that, phenomenally, contraction is a regular and pervasive feature of human visual experience.

The contraction shown in Figure 1 accords with the size–distance invariance hypothesis (SDIH). This hypotheses, which is well-confirmed empirically [2,3], is represented in the figure under the assumption that phenomenal visual angles coincide with physical visual angles (that is, that visual direction is accurately perceived). The SDIH does not by itself predict what size an object will appear to have. Rather, it specifies that the apparent size of an object is a product of the object’s registered or perceived visual angle and its registered or perceived distance. The terms *registered* and *perceived* distinguish values that may be (a) nonconsciously registered and processed by the visual system as opposed to (b) being phenomenally available in consciousness [71]. If distance and visual angle are accurately registered, then full phenomenal constancy results. If distance is under-represented, then a contracted visual space occurs. The more distance is under-represented, the greater the contraction. This relation is also found in Wagner’s Vector Contraction Model as mentioned above.

Three basic principles can be deduced from Hatfield’s Perspective-Based Model. First, lines parallel to the y-axis (the depth dimension) of physical space appear to converge at a vanishing point at a finite apparent distance (*v*) away from the observer in visual space. Secondly, visual direction is preserved; that is, the angular deviation of a point from straight ahead in visual space is the same as in physical space. Thirdly, after the transformation, which is a form of perspectival projection along straight lines, visual space is Euclidean and follows the geometry of the size–distance invariance hypothesis. In the present context, this means that (as in Figure 1) squares in physical space would, in visual space, contract to trapezoids whose angles sum to 360°. This later assumption is not required by the theory, but it fit Hatfield’s intuition and made the theory more mathematically tractable. We retain this assumption for now. Based upon these three assumptions, it is possible to derive equations that show how coordinates in physical space transform into coordinates in visual space. (See Appendix A for this derivation.) 

For a point in physical space with coordinates (*x*, *y*, *z*) (width, depth, height) the coordinates in visual space (*x*′, *y*′, *z*′) after the perspective transformation are determined by the following equations:

(2) $$x^{\prime }=\frac{xv}{y+v}$$

(3) $$y^{\prime }=\frac{yv}{y+v}$$

(4) $$z^{\prime }=\frac{zv}{y+v}$$

Some readers may notice that these equations are not new. They closely correspond to the equations Gilinsky [40] introduced for perceived size and distance in her important *Psychological Review* paper. Gilinsky derived her theory from a portion of Luneburg’s hyperbolic geometry model. Fry [72] and Smith [73] soon questioned whether her theory really corresponded to Luneburg’s. They observed that Gilinsky’s theory really assumed that visual space was Euclidean with zero curvature, rather than Luneburg’s assumption of constant negative curvature. In fact, Gilinsky derives her equations in a second way based on the laws of perspective, and this derivation clearly assumes that visual space is Euclidean.

Recently, Ooi and He [69] have revived interest in Gilinsky’s work. They show that Gilinsky’s theory should be associated with misperception of ground surface slant. As Equation (4) implies, vertical surfaces should also appear to converge to the vanishing point, and this should correspond to a perception that the ground is slanted upward. Hatfield [42,43] also made this prediction from his model. Ooi and He empirically confirmed their predictions about ground slope perception. As they predicted, the degree of error depended on the height of the observer, since a taller observer has a more distant vanishing point. The degrees of ground slope misperception are well predicted by the equations presented above.

Erkelens [35,36,37,38,39] has also proposed a perspective-based model for visual space. Erkelens generalizes Gilinsky’s model and uses a single parameter, a variable vanishing point, to account for numerous phenomena such as Hillebrand [16] and Blumenfeld’s [17] parallel alleys, and findings on the relationship between estimated size and distance.

Hatfield, Erkelens, and Gilinky‘s models have the odd feature of positing a bounded space that is simultaneously Euclidean. The space is bounded because visual space has a maximun distance, *v*. However, unlike hyperbolic or spherical spaces which are bounded spaces by their very nature, Euclidean spaces are generally not bounded. A second thing to note is the similarity between the effects of Equation (1) with a variable compression parameter and those of Equations (2)–(4). Both predict a systematic decline in perceived size as a function of distance from the observer.

A perspective transformation would affect the perceived shape (appearance) of an object that is physically square (as seen along a ground plane in front of a standing observer). A physical square medially centered in an in-depth plane should look like an isosceles trapezoid with the side of the figure nearest to the observer seeming longer than the side farthest away. Assuming that visual space is Euclidean after the transformation, the interior angles of this trapezoid should sum up to 2π or 360°; however, the interior angles closer to the observer should seem perceptually smaller than the interior angles that are farther away. If the space is Euclidean, each of the near angles should be smaller than 90° and each of the far angles should seem greater than 90°. 

Under these assumptions, if squares were placed on the ground in front of a standing observer, the observer would look down at the nearer squares and see the figure in a more frontal orientation, while distant squares would be more oriented in depth. For this reason, a perspective-based transformation would have a greater effect on the perceived shape of distant figures than near ones, and the effects of perspective on the perceived size of distant squares should be more pronounced than for near ones.

Mathematical simulations using Excel confirm this conclusion. In the simulation, squares placed on the ground at various distances from the observer were transformed into visual space using Equations (2)–(4), and the interior angles of the squares after the transformation were determined. In the simulation, for all squares, the interior angles closer to the observer within a given square were smaller (less than 90°) than the interior angles at the more distant end of the square (greater than 90°). The interior angles of near squares did not differ from 90° as much as the interior angles of more distant squares. The deviation of distant angles from 90° was greatest when the vanishing point distance *v* used in the simulation was small.

## 4. The Present Experiments

The present work presents two experiments that test the predictions of the simple affine and perspective-based models for visual space, including the assumption that the transformations are Euclidean. Standing observers were asked to judge the size of the interior angles of five squares placed on the ground at various distances away and centered about the median plane. The two models of visual space make different predictions about these judgments.

Simple affine models predict that the angles should still sum up to 360°, since they assume that visual space has a contracted Euclidean structure after the transformation. If the sides of the square are parallel to the frontal and in-depth dimensions relative to the observer, the affine model predicts that the 90° corner angles should remain 90° after the square is perceptually transformed into a rectangle. (Once again, this prediction is only true under the assumptions given; if the squares were viewed from some oblique angle, we would expect the size of the angles to change.)

Perspective-based models also predict that the four angles of the square should sum up to 360°, under the assumption that visual space is Euclidean after the transformation. However, the angles formed with the side nearer to the obsever should be slightly smaller than 90°, and the angles formed with the side farther from the observer should be slightly greater than 90°. The prediction that near angles should seem smaller than far angles differentiates perspective-based models from simple affine transformation models. The Vector Contraction model makes predictions similar to the perspective-based models. Finally, if the angles summed up to more than 360°, this would be evidence that the transformation is non-Euclidean with positive curvature (elliptic). A sum of less than 360° suggests a negative curvature (hyperbolic).

Past research on spatial perception of size, distance, angle, and area have used four different instruction types to communicate the meaning of various target metric properties to participants: objective, perspective, apparent, and projective instruction types. *Objective instructions* ask the observer to match or estimate the actual physical size of the stimulus; if they respond accurately, all angles should be reported as 90° for the stimuli described. *Perspective instructions* ask observers to take into account the laws of perspective when making judgments; they might be reminded that railroad tracks appear to converge to a vanishing point and instructed to correct for this apparent shrinkage. *Apparent instructions* ask observers to alter a comparison until it subjectively “appears” or “looks” to be the same size as the standard. *Projective instructions* require observers to ignore depth information altogether and estimate the amount of their visual field subtended by the stimulus; observers might be asked to take an “artist’s eye view” in which, comparing their visual experience to a mental canvas, they estimate how much of this canvas the stimulus covers. Wagner [1] performed a meta-analysis of 413 data sets of metric judgments from the direct estimation literature and subsequently performed a meta-analysis of 119 data sets from the size constancy literature [2], finding each time that apparent and objective instruction-types were the most commonly used .

The present research collects estimates of the sizes of angles under apparent size instructions. These instructions most closely correspond to the subjective visual space that is our target. Hatfield [43] and Granrud [74], for example, have argued that objective instructions add a cognitive component to judgments, while apparent instructions more closely correspond to phenomenal experience.

## 5. Experiment 1

Experiment 1 represents our initial investigation of the perceived size of the four angles in each of five squares.

### 5.1. Method

**Participants.** Thirty undergraduate students (28 females and 2 males ranging from 18 to 22 years old) from Wagner College participated. Participation was voluntary. The College’s Human Experimental Review Board approved the experiment. All participants gave their informed consent. They were allowed to withdraw at any time, but none did.

**Materials and experimental layout.** Five squares were laid out on the floor of a corridor using 2.54 cm wide silver duct tape. The sides of each square were 0.5 m in length (external dimensions). Participants stood just behind a line on the floor. The squares were at various distances from the participant one behind the other, with their centers aligned with the participant’s line of sight (which was perpendicular to the line on the floor). They were oriented with two sides parallel to the participant’s frontal plane and the remaining two sides parallel to the participant’s median plane (oriented in-depth relative to the observer). The center of the near edge of the first square was 0.5 m from the participant, the second was 1.5 m away, the third was 2.5 m away, the fourth was 4 m away, and the fifth was 8 m away. 

Angle estimates were obtained using a “compass” modeled after Proffitt’s [75] “visual matching device” that he used for slant estimation. A piece of cardboard was cut into a circle and half was painted black, the other half yellow. Another piece of cardboard was cut in the shape of a half circle with a raised tab on one end of the arc. The two pieces were attached to each other loosely enough to allow the half circle to be rotated until the desired amount of yellow was showing, representing the participant’s perceived angle size. A circular protractor on the back of this device allowed the experimenter to record the numeral value of the angle estimate. Participants never saw the back of the compass or the protractor, and so received no feedback about their estimates.

**Procedure.** Each of the 30 participants performed the experiment individually. They stood just behind the line facing the five squares on the floor. Each was given the compass and told that he or she needed to adjust it in order to match the apparent size of all 20 interior angles of the squares. Half began with the nearest square, half with the most distant square. Participants made estimates working clockwise around the interior angles of each square and then went on to estimate the angles of the next square. They held the device in front of them with the wedge specifying the angle in their frontal plane. Sometimes they moved the compass slightly to the left or right of the median plane, however.

After each adjustment, the experimenter retrieved the compass from the observer and used the protractor on the back to record the adjusted angle. Participants were not told the numerical value of their estimate. The compass was reset to 0° to begin the next judgment.

Participants were given instructions to judge the apparent rather than objective size of each angle. The exact instructions were:

You see five squares in front of you at different distances away. Although we all know that the interior angles for each corner of the square are physically 90 degrees, they might or might not appear or “look” that size subjectively. Please adjust this compass until it matches the apparent size of each of the interior angles of the squares. Start with the bottom right angle of the nearest [or most distant] square and go clockwise around the square, and then proceed to the next square. In between each adjustment, I will take the compass from you and record your answer. Please don’t turn the compass over, since we want you to rely on your subjective impressions, not the numbers on the back. Remember, we don’t want you to report the actual physical size of the angle, but we want you to tell us how large each angle looks or appears.

After completing their estimates, participants were thanked and debriefed about the purpose of the experiment.

### 5.2. Results

The present experiment examined estimates of the size of the interior angles of squares as a function of distance to each square and the location of angles within a square. The dependent variable was the average angle estimate and the three independent variables were the distance from the participant to the square (which we will call *distance*), whether an angle within a square was on the side nearer to the observer or the side more distant (which we will call *near* vs. *far location*), and whether the angle was on the right or left side of the square relative to the observer (which we will call *right* vs. *left location*). To test the effects of these independent variables on angle estimates, a three-way, repeated-measures Analysis of Variance was performed.

Although the mean angle estimate was somewhat larger for angles on the right side of the squares (M = 86.17) than the left (M = 85.03), this difference was not significant, *F*(1,29) = 3.29, *p* = 0.08, η_p_^2^ = 0.102. In addition, the left vs. right location variable did not interact significantly with either distance to squares or near vs. far location within a square and need not be considered further.

**Figure 2:**
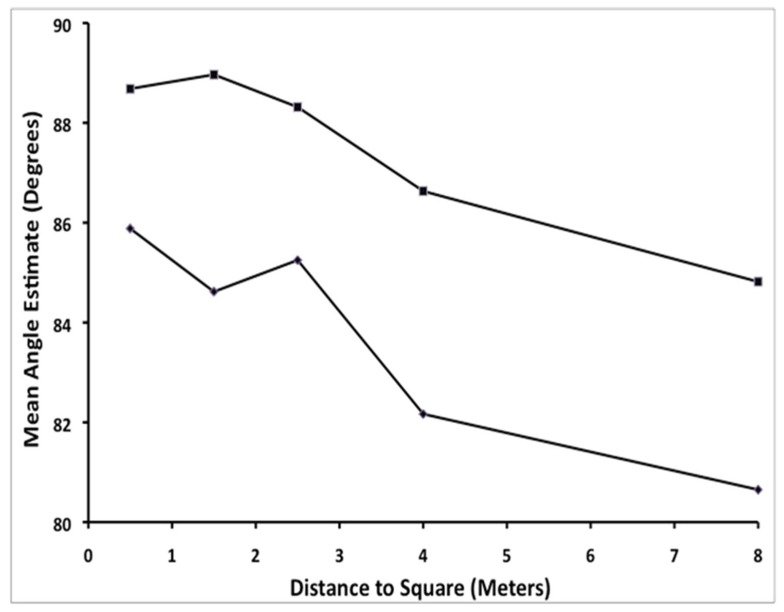
Mean angle estimate (in degrees) as a function of distance from the observer to each square (in meters) and location of angle within a square for Experiment 1. Diamonds (bottom line) indicate mean angle estimates for near angles and squares (top line) indicate mean angle estimates for far angles within a square.

There were significant main effects on angle estimates for both distance to the square and near vs. far location. Table 1 shows means and standard errors for angles estimates as a function of distance both overall and separately for near vs. far location. Figure 2 also illustrates these effects. The figure shows the mean angle estimate as a function of distance to each square and near vs. far location of the angle within a square. Notice that mean angle estimates are always smaller for near angles than for angles at the far end of a square for all distances, a result that is consistent with the predictions of Hatfield, Erkelens, and Gilinsky’s perspective models. Also notice that the deviation of angle estimates from 90° increases with increasing distance to the square. However, contrary to what any Euclidean model would predict, both near and far angle estimates are less than 90° on average, hence sum up to less that 360°. In addition, the table shows that the standard error of the estimates increases with distance, indicating that judgment precision goes down as distance to the square increases.

The repeated measures ANOVA revealed that the mean angle estimate was significantly greater for near squares and smaller for more distant squares, *F*(4,116) = 4.21, *p* = 0.003, η_p_^2^ = 0.13. In addition, mean angle estimates for angles near to the observer (M = 83.71) were significantly smaller on average than those more distant from the observer (M = 87.43) within the same square, *F*(1,29) = 10.00, *p* = 0.004, η_p_^2^ = 0.26. On the other hand, the interaction effect between distance and near vs. far location on angle estimates was not significant, *F*(4,116) = 0.34, *p* > 0.05.

**Figure 3:**
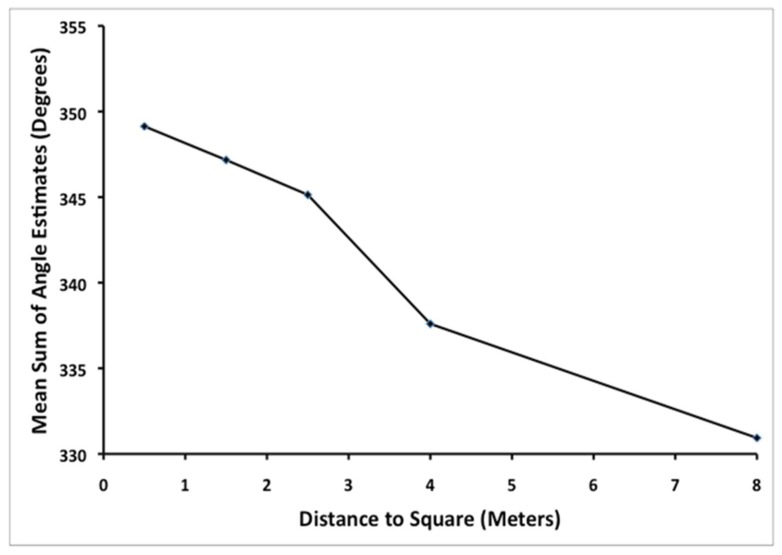
Mean sum of the angle estimates (in degrees) for each square as a function of distance (in meters) to the square from the observer to the square in Experiment 1.

Euclidean geometry would predict that the angles of a quadrilateral should always sum up to 360°, while a sum of less than 360° would be consistent with a geometry of negative curvature. To examine these predictions, each participant’s angle estimates for a given square were summed. Figure 3 shows the average sum of the angle estimates as a function of distance to the square. Notice that the sum of the angle estimates is always less than 360° and that this sum decreases significantly as a function of distance from the participant to the square, *F*(4,116) = 4.21, *p* = 0.003, η_p_^2^ = 0.13.

### 5.3. Discussion

The results of this experiment showed that the apparent size of the interior angles of squares do not match their physical size. In particular, interior angles nearer to the observer are perceived to be consistently and significantly smaller than angles farther away from the observer within the same square. In addition, the estimated apparent size of the interior angles significantly decreases as physical distance from the observer to the square increases. Finally, the sum of the angle estimates across the four interior angles of each square is consistently less than the 360° predicted by Euclidean geometry. The sum of the angles deviates increasingly from the Euclidean prediction as the distance from the observer to the square increases.

However, Experiment 1 suffers from procedural issues that call for improvement. First of all, only one judgment was collected for each angle and all estimates were based only on ascending adjustments that began with the compass set at 0°. Using multiple judgments per angle would allow for more precision in estimating perceived angular size. More importantly, the use of only ascending trials could have resulted in systematic response bias (either errors of expectation or errors of habituation) that could result in general under- or over-estimation of angular size.

This could influence the interpretation of the data. Some might argue that visual space could still be Euclidean, but this procedure led to an error of expectation where judgments systematically undershoot the true perceptual value. If this were true, however, it should have produced a specific pattern in the data. If the space were Euclidean with a constant error we would still expect that a decline in the estimated size of the near angles in a square should be linked to a corresponding increase in the estimated size of the more distant angles within the square. This pattern is contrary to what we found with our data. To redress possible response bias and to increase precision in angle estimates, Experiment 2 will use both ascending and descending adjustment trials. 

Another weakness of Experiment 1 is that observers were asked simply to estimate the “apparent size” of each angle. The undergraduates in this study may have estimated apparent angle size accurately, or they may have allowed their judgments to mean something different. For example, the projective size on the retina of the squares and the angles that compose them will decline as distance increases and the angle of regard becomes less frontal. If participants interpreted perceived size of an angle as being how much of the retina the angle takes up, their estimates would produce a pattern of results similar to what we report here.

This ambiguity in the meaning of apparent size has a long history. Phenomenologists such as Hatfield [43] and empirical researchers such as Granrud [74] find that apparent length or area declines with increasing distance in accordance with perspective-based models. However, past research using apparent size instructions has not always been consistent with this pattern. Wagner [2,3] performed a meta-analysis of 125 size constancy data sets from published works over the last century. This analysis found that apparent size instructions led to accurate size constancy on average with as many studies reporting underconstancy as those reporting overconstancy. Wagner [2] suggests that apparent size instructions might produce a range of results that average out to constancy because observers may interpret the instructions in various ways, some interpreting the instructions as requesting projective size, some perspective size, and some objective size.

To make estimates less ambiguous, Experiment 2 uses instructions that ask observers to base their estimates on the apparent number of degrees that each angle spans. Directing participants to report the apparent number of degrees invites them to focus on this specific aspect of how an angle appears; whereas, just asking them to report its “apparent size” might allow them to base their judgments on other aspects of the stimulus. Experiment 2 aims to clarify the meaning of the participants’ judgments and to determine if the phenomena revealed in Experiment 1 are replicable.

## 6. Experiment 2

### 6.1. Method

**Participants.** The thirty participants ranged from 18 to 30 years old. Participation was voluntary, and all participants gave their informed consent.

**Materials and experimental layout.** As in Experiment 1, five squares were laid out on the floor of a corridor using 2.54 cm wide silver duct tape. The sides of each square were 0.5 m in length (external dimensions). Participants stood just behind a line on the floor. The squares were at various distances from the participant one behind the other, with their centers aligned with the participant‘s line of sight. They were oriented with two sides parallel to the participant’s frontal plane and the remaining two sides parallel to the participant’s median plane (oriented in-depth relative to the observer). The center of the near edge of the first square was 0.5 m from the participant, the second was 1.5 m away, the third was 2.5 m away, the fourth was 4 m away, and the fifth was 8 m away. 

Angle estimates were obtained using the same “compass” modeled after Proffitt’s [75] “visual matching device.” As before, participants received no feedback about the size of their estimates.

**Procedure.** Each of the 30 participants performed the experiment individually. They stood just behind the line facing the five squares on the floor. Each was given the compass and told that he or she needed to adjust it in order to match the apparent size of all 20 interior angles of the squares. Half began their estimation with the nearest square, half with the most distant square. They made estimates working clockwise around the interior angles of each square and then went on to estimate the angles of the next square. The participants held the device in front of them with the wedge specifying the angle in their frontal plane. Sometimes they moved the compass slightly to the left or right of the median plane, however.

After each adjustment, the experimenter retrieved the compass from the observer and used the protractor on the back to record the estimate. Participants were not told the numerical value of their estimate. For each angle, participants performed both ascending trials, where the compass was reset to 0° prior to making the estimate, and descending trials, where the compass was reset to 180° prior to making the judgment. Half of the participants performed the ascending trial before the descending trial, and half performed the descending trial first.

Participants were given instructions to judge the apparent angular size of the angle. The exact instructions were:

You see five squares in front of you at different distances away. Although we all know that the interior angles for each corner of the square are physically 90 degrees, they might or might not appear or “look” that angular size subjectively. Please adjust this compass until it matches the apparent number of degrees of each of the interior angles of the squares. For each angle we will have you do two adjustments, one where the compass angle is initially zero and must be adjusted outward and one where the compass angle is initially 180° and must be adjusted inward. In between each adjustment, I will take the compass from you and record your estimate. Please don’t turn the compass over to see the numbers, since we want you to rely on your subjective impressions, not the numbers on the back. Start with the bottom right angle of the nearest [or farthest] square and go clockwise around the square, and then proceed to the next square. Remember, we don’t want you to report the actual physical number of degrees of each angle nor the area occupied by the angle, but we want you to tell us how many degrees each angle looks or appears to span.

After completing their estimates, participants were thanked and debriefed about the purpose of the experiment.

### 6.2. Results

The dependent variable was the average angle estimate and the four independent variables were the distance from the participant to the square (which we will call *distance*), whether an angle within a square was on the side of the square nearer to the observer or the side more distant (which we will call *near* vs. *far location*), whether the angle was on the right or left side of the square relative to the observer (which we will call *right* vs. *left location*), and whether the trial involved an ascending or descending judgment (which we will call *trial*). To test the affects of these independent variables on angle estimates a four-way, repeated-measures Analysis of Variance was performed.

Experiment 2 provided more precise and powerful results than the initial experiment, and more effects proved to be significant. In this experiment, both right vs. left location and trial significantly affect judgments. Once again, the mean angle estimate was somewhat larger for angles on the right side of the squares (M = 86.84, std. error = 0.83) than the left (M = 85.50, std. error = 0.70), but in the second experiment this difference in means proved to be significant, *F*(1,29) = 14.37, *p* = 0.001, η_p_^2^ = 0.33. In addition, ascending trials produced significantly smaller mean angle estimates (M = 85.22, std. error = 0.80) than descending trials (M = 87.12, std. error = 0.70), *F*(1,29) = 127.05, *p* < 0.001, η_p_^2^ = 0.81. Thus, the data show a strong error of expectation effect. In addition, there were several significant higher-order interaction effects. For example, there were significant three-way interactions between distance, left vs. right, and trial, *F*(4,116) = 4.04, *p* = 0.004, η_p_^2^ = 0.12, and between near vs. far, left vs. right, and trial, *F*(1,29) = 7.08, *p* = 0.01, η_p_^2^ = 0.19. These interactions do not bear on this study’s main hypotheses and so will not be discussed further.

**Figure 4:**
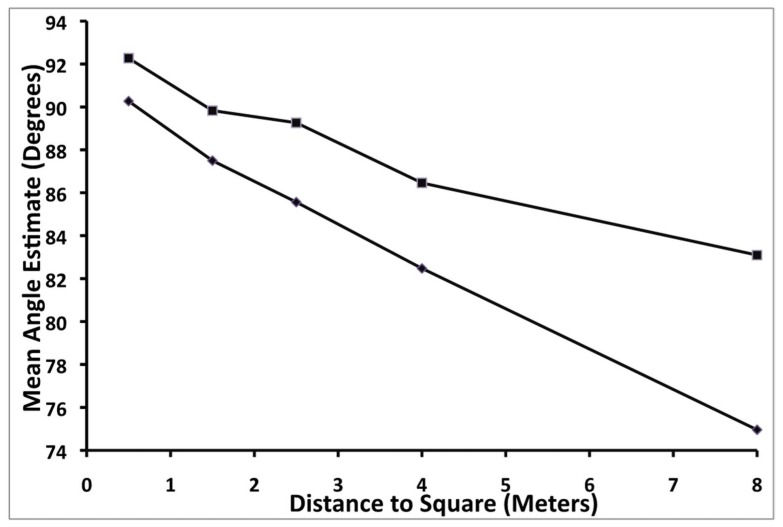
Mean angle estimate (in degrees) as a function of distance from the observer to each square (in meters) and location of angle within a square in Experiment 2. Diamonds (bottom line) indicate mean angle estimates for near angles and squares (top line) indicate mean angle estimates for far angles within a square.

Of more theoretical importance, there were significant main effects on angle estimates for both distance to the square and near vs. far location within a square. Table 2 shows means and standard errors for angles estimates as a function of distance both overall and separately for near vs. far location. Figure 4 also illustrates these effects. The figure shows the mean angle estimate as a function of distance to each square and near vs. far location of the angle within a square. Once again, mean angle estimates are always smaller for near angles within a square than for angles at the far end of a square for all distances, a result that is consistent with the predictions of Hatfield, Erkelens, and Gilinsky’s perspective-based models. Also notice that angle estimates decline with increasing distance to the square. In addition, the table shows that the standard error of the estimates tends to increase with distance, indicating that judgment precision goes down as distance to the square increases. Standard errors also tend to be larger for near angles within each square than for far angles, indicating greater precision for far angle estimates than near angle estimates.

The repeated measures ANOVA (using the conservative Greenhouse-Geisser correction for a significant Mauchly Test of Sphericity) revealed that the mean angle estimate was significantly greater for near squares and smaller for more distant squares, *F*(2.36,68.56) = 18.09, *p* < 0.001, η_p_^2^ = 0.38. Sidak post hoc tests showed that all angle estimates differ significantly from each other as a function of distance except that angle estimates for the second square (1.5 m from the observer) do not differ significantly from those of the first (0.5 m) or third (2.5 m) squares. In addition, within the same square, mean angle estimates for angles near to the observer (M = 84.15) were significantly smaller on average than those more distant from the observer (M = 88.18), *F*(1,29) = 74.27, *p* < 0.001, η_p_^2^ = 0.72. Note that this result has very large *F* statistics and effect sizes. Unlike the first experiment, the interaction effect between distance and near vs. far location on angle estimates was significant (using the Greenhouse-Geisser correction for a significant Mauchly Test of Sphericity), *F*(2.68,77.63) = 6.51, *p* = 0.001, η_p_^2^ = 0.18. This small interaction effect reveals that the difference between mean near and far estimates grows somewhat with increasing distance from the observer.

**Figure 5:**
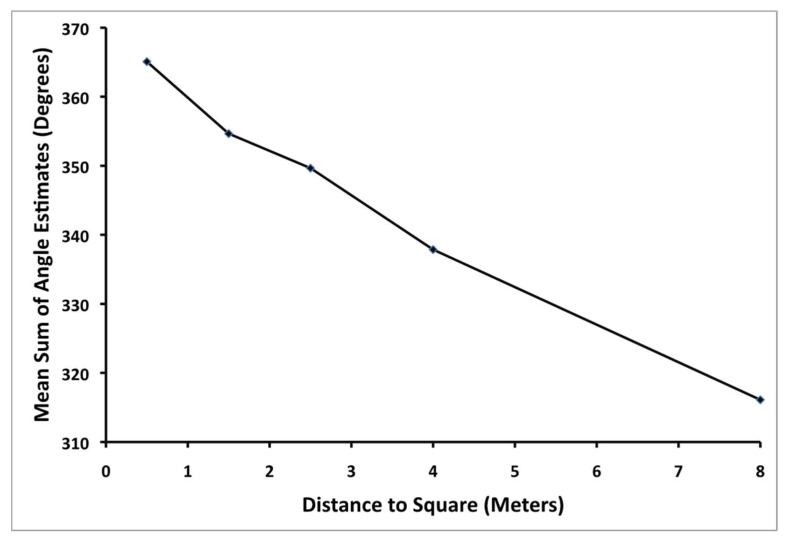
Mean sum of the angle estimates (in degrees) for each square as a function of distance (in meters) to the square from the observer to the square in Experiment 2.

Once again, Euclidean geometry would predict that the sum of the angles of a quadrilateral should always sum up to 360°, while a geometry of negative curvature would predict that the angles should sum up to less than 360°. To examine these predictions, each participant’s angle estimates for a given square were summed. Figure 5 shows the average sum of the angle estimates as a function of distance to the square. Notice that the sum of the angle estimates is slightly greater than 360° for the nearest square but less than 360° for all others and that this sum decreases significantly as a function of distance from the participant to the square, *F*(2.36, 68.56) = 18.09, *p* < 0.001, η_p_^2^ = 0.38. This result is consistent with a slight positive curvature to visual space very near to the observer and increasingly negative curvature with increasing distance. This pattern of change in curvature is strikingly similar to a number of previous works [76,77,78,79].

One early reader of this paper suggested an alternate interpretation of our data. The reader suggested that location of angles within the square was unimportant and that judgments of “near” and “far” angles do not differ beyond the effects of differences in their egocentric distances. In other words, the reader suggested that angle estimates simply decrease with increasing distance from the observer, and whether the angle is located on the near or far sides of the square does not matter above and beyond this effect. However, this alternative explanation is not consistent with the data. Since the far angles are 0.5 m farther from the observer than the near angles, this alternative explanation would predict that far angle estimates should be smaller than near angle estimates, but the data shows the opposite trend. Far angles estimates are consistently larger than near angle estimates. In fact, plotting mean angle estimates as a function of distance to the angle itself (instead of to the near edge of the square) would result in the top line of Figure 4 moving to the right 0.5 m. Instead of the difference in response to near vs. far angles going away, this way of displaying the data actually magnifies the differences in response to the different angle locations in a square.

### 6.3. Discussion

The results of Experiment 2 are largely consistent with those of Experiment 1. Near angles within a square are consistently estimated to be significantly smaller than far angles at all distances from the observer, in accord with perspective-based models once again. The sum of the angle estimates of a square declined with increasing distance once more. However, in the second experiment, the sum of the angles was slightly greater than 360° for the nearest square, but smaller than 360° for all other squares. Given that there was a small but significant error of expectation in Experiment 2, it is possible that the estimates of the first study that relied only on ascending judgments might be slightly too low. So, even this small discrepancy in the data is understandable.

The inclusion of multiple judgments for each angle and the use of instructions that clarified that observers should judge the apparent number of degrees of each angle had a second beneficial effect. The second experiment produced more powerful results and impressive magnitudes of effect.

The two experiments taken together show that we have discovered a reliable and replicable phenomenon. Near angles within a square are reliably smaller than far angles, the judged size of angles reliably decreases with increasing distance, and the sum of the angle estimates within a square are reliably lower than the Euclidean value of 360° for all but the nearest stimuli.

## 7. General Discussion

Both of these experiments showed that the apparent size of the interior angles of squares do not match their physical size. In particular, interior angles nearest to the observer are perceived to be consistently and significantly smaller than angles farther away from the observer within the same square. In addition, the estimated apparent size of the interior angles significantly decreases as physical distance from the observer to the square increases. Finally, the sum of the angle estimates across the four interior angles of each square is consistently less than the 360° predicted by Euclidean geometry. The sum of the angles deviates increasingly from the Euclidean prediction as the distance from the observer to the square increases.

These results can be compared to the predictions of the simple affine and perspective-based models described earlier. The data are consistent with some of these predictions; however, neither of the models can account for all of the data.

The simple affine model is least consistent with our data. Affine models would predict that the perceived sum of the angles of the squares should remain 360° and that the interior angles of squares in the specific orientation we present should continue to be perceived as being 90° after the affine transformation. Angles nearer to the observer should be perceived as the same size as angles farther from the observer within a square. None of these predictions is supported by our data. The sum of the estimated size of the angles of a square is almost always less than 360°, individual angles are estimated to be almost always less than 90°, and near angles within a square are perceived as smaller than far angles.

The simple affine model fails, in part, because it clings to a number of simplifying assumptions. It describes visual space using a Cartesian coordinate system and posits that the y-dimension (in-depth) of this space is perceptually compressed by the same amount no matter how far to the left or right of straight ahead a stimulus is. Yet, the visual experience of an individual is probably more akin to a polar coordinate system. Two stimuli with the same x-coordinate and differing y-coordinates are only fully seen in-depth with respect to the observer when the x-coordinate is zero, and as the x-coordinate increasingly deviates from zero, the two stimuli in this example are seen at increasingly frontal orientations relative to the observer. If visual space is truly compressed in the in-depth dimension relative to the observer, the compression at a given distance away should be conceived of as taking place along a circle centered at the observer (or perhaps more accurately, the compression takes place along the visual horopter), not in terms of arbitrary Cartesian coordinates. Accordingly, consideration should be given to Wagner’s [3,33] Vector Contraction Model, which conceives of compression in these polar terms. The Vector Contraction Model predicts that the near angles of a square should be seen as smaller than the far ones; however, it would still be inconsistent with our data in predicting that the far angles should be greater than 90°.

In the present study, Hatfield, Erkelens, and Glinsky’s perspective-based models have more success than the simple affine model in accounting for our data. Perspective-based models predict that the near angles within a square should be perceived to be smaller than the more distant angles within a square, just as our data shows. In addition, perspective-based models predict that, under our stimulus conditions, the near angles should deviate perceptually more from 90° for far squares than for near ones, just as our data shows. However, past perspective-based models have made the simplifying assumption that visual space is Euclidean after the perspective transformation. This implies that the sum of the angles of a quadrilateral should sum to 360° and that the angles most distant from the observer within a square should be seen as being greater than 90° and that they should seem increasingly large with increasing physical distance to the square. These later predictions are inconsistent with our data. Thus it would appear that proponents of perspective-based models are largely successful, but should question the simplifying assumption that visual space is Euclidean.

The sum of the interior angles of the squares was almost always less than 360° at all distances from the observer. In addition, the sum of the angles declined with increasing distance from the observer. These observations are consistent with past work that shows that the curvature of visual space might not be constant. Ivry and Cohen [77]) and Koenderink, van Doorn, and Lappin [78,79] have presented evidence that the curvature of visual space is not constant and that its curvature becomes increasingly negative with increasing distance from the observer. Our data support the idea that visual space may have increasingly negative curvature with increasing distance from the observer under the specific conditions of our study. Our results, otherwise consistent with perspective-based models, thus contribute converging confirmation that visual space exhibits negative curvature under a variety of conditions.

Alternatively, our initial technique may not have eliminated all possible response biases. Angle estimates may decline with size simply because the projective size of stimuli shrink as distance from the observer increases; so, observers may think the angles shrink along with the stimuli’s projective size. Such cognitive shrinkage would be more likely with a numeric estimation task than the matching task used here. To avoid this potential problem, the second experiment explicitly used instructions emphasizing that observers should base their estimates on the apparent number of degrees that each angle spans.

In summary, the perspective-based model is better supported by our results than the affine model. However, consistent with some previous findings, perspective-based models may need to incorporate non-Euclidean transformations of physical space into their descriptions. Only systematic parametric work that describes the metric of visual space throughout the visual field will be able to offer a closer specification of the geometry or geometries of visual space in relation to various instructions and stimulus conditions.

## Figures and Tables

**Table 1:** Means and standard errors (in parentheses) for angles estimates as a function of distance both overall and separately for near vs. far location.

Distance to Square	Angles Estimated		
	Overall Estimate	Near Estimates	Far Estimates
0.5 m	91.27 (0.57)	90.27 (0.64)	92.27 (0.60)
1.5 m	88.66 (1.13)	87.50 (1.23)	89.83 (1.12)
2.5 m	87.41 (1.09)	85.57 (1.28)	89.26 (1.04)
4.0 m	84.47 (1.25)	82.46 (1.69)	86.46 (1.04)
8.0 m	79.03 (1.86)	74.96 (2.42)	83.10 (1.42)

**Table 2:** Means and standard errors (in parentheses) for angles estimates as a function of distance both overall and separately for near vs. far location.

Distance to Square	Angles Estimated		
	Overall Estimate	Near Estimates	Far Estimates
0.5 m	87.28 (0.85)	85.88 (1.03)	88.68 (1.08)
1.5 m	87.79 0.70)	84.62 (0.85)	88.97 (0.99)
2.5 m	87.78 (0.88)	85.25 (1.50)	88.32 (1.05)
4.0 m	84.40 (1.34)	82.17 (1.53)	86.63 (1.63)
8.0 m	82.73 (1.89)	80.65 (2.25)	84.82 (2.13)

**Figure A1:**
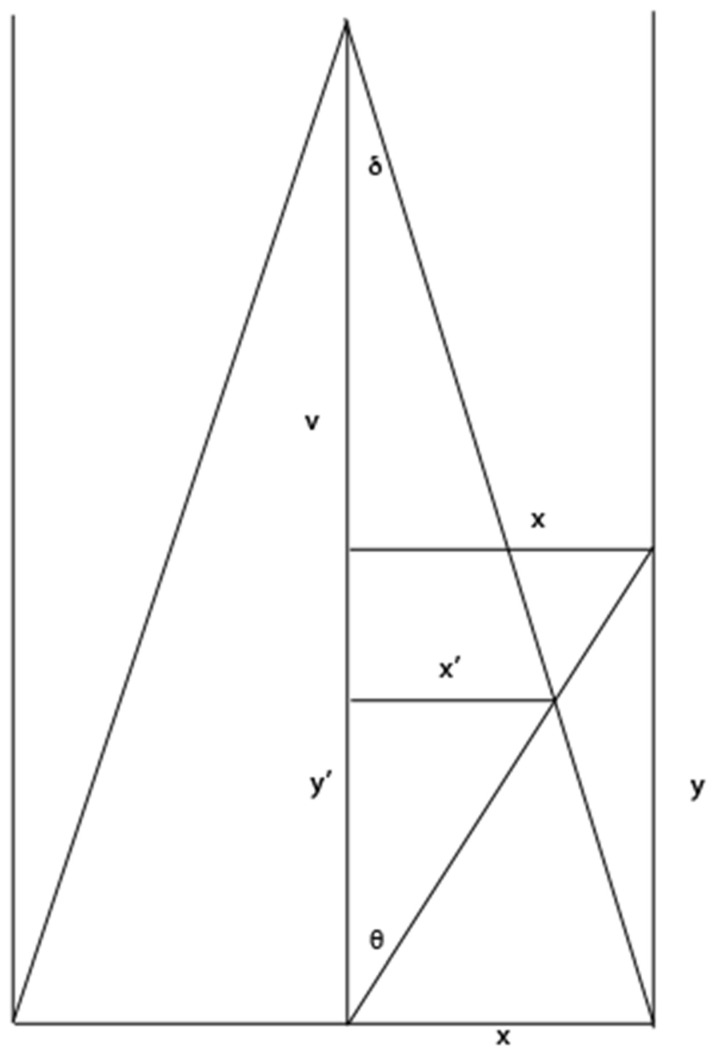
Diagram illustrating parallel alleys transformed according to Hatfield’s Perspective-Based model.

## Appendix A

According to the linear perspective, physically parallel lines converge to a vanishing point perceptually. The distance to the vanishing point is *v* and the convergence angle for the vanishing point for one side of the triangle is *δ* (for the whole figure it is 2*δκ*). According to the Hatfield’s model, the visual direction to objects is preserved. That is, the visual direction to an object (*θ*) in visual space is the same as it is in physical space. If there are two parallel alleys in physical space oriented along the observer’s line of sight, the *x* is the left–right distance from the observer to the walls of the alley and *y* is how far a point recedes in depth away from the observer’s plane. The corresponding coordinates in visual space are *x*′ and *y*′. Figure 1 shows this three-dimensionally, but imagine we were looking down on the situation from the top, you’ll see the two-dimensional schematic depicted in Figure A1. (We’ll get back to the third dimension in a while.)

This gives us the following initial relations:

(A1) $$\tan\theta =\frac{opposite}{adjacent}=\frac{x}{y}=\frac{x^{\prime }}{y^{\prime }}$$

(A2) $$\tan\delta =\frac{x}{v}=\frac{x^{\prime }}{v-y^{\prime }}$$

From Equation (A1),

(A3) $$x^{\prime }=\frac{xy^{\prime }}{y}$$

From Equations (A2) and (A3) we have

(A4) $$x^{\prime }=\frac{x\left(v-y\prime \right)}{v}=\frac{xy\prime }{y}$$

This in turn leads to

(A5) $$y^{\prime }=\frac{xy\left(v-y\prime \right)}{xv}=\frac{y\left(v-y\prime \right)}{v}=\frac{yv-yy\prime }{v}$$

(A6) $$vy^{\prime }=yv-yy\prime$$

(A7) $$yv=yy^{\prime }+vy^{\prime }=y\prime \left(y+v\right)$$

(A8) $$y^{\prime }=\frac{yv}{\left(y+v\right)}$$

Combining Equations (A3) and (A8) yields

(A9) $$x^{\prime }=\frac{xy\prime }{y}=\frac{x\left[\frac{yv}{\left(y+v\right)}\right]}{y}=\frac{\frac{xyv}{\left(y+v\right)}}{y}=\frac{xyv}{y\left(y+v\right)}=\frac{xv}{\left(y+v\right)}$$

Now, it’s time to add the third dimension. If you look at the lines of perspective from the side, you see a geometrically equivalent diagram where *z* (height) takes the place of *x* (width). Thus, perceived height (*z*′) is found by substituting *z* for *x* in to Equation (A9) to yield

(A10) $$z^{\prime }=\frac{zv}{\left(y+v\right)}$$

Now, in the situation we have described, *x*, *y*, and *z* can be thought of as defining coordinates of a point in a Cartesian space. So, the equations we have developed can be thought of as describing the coordinates in visual space that correspond to a location in physical space. That is, for a position, P, in physical space defined by the coordinates *x*, *y*, *z* and a vanishing distance *v*, the coordinates of the point in visual space (P′) corresponding to P would be

(A11) $$x^{\prime }=\frac{xv}{\left(y+v\right)}$$

(A12) $$y^{\prime }=\frac{yv}{\left(y+v\right)}$$

(A13) $$z^{\prime }=\frac{zv}{\left(y+v\right)}$$

In truth, we have assumed Euclidean geometry to get to this point, since we have used Euclidean trigonometry to do the derivation. Keeping with this assumption, the distance between the two points in visual space (*x’*_1_, *y’*_1_, *z’*_1_) and (*x’*_2_, *y’*_2_, *z’*_2_) that correspond to two points in physical space (*x*_1_, *y*_1_, *z*_1_) and (*x*_2_, *y*_2_, *z*_2_) would be

(A14) $$d^{\prime }=\sqrt{{\left(x\prime_{1}-x\prime_{2}\right)}^{2}+{\left(y\prime_{1}-y\prime_{2}\right)}^{2}+{\left(z\prime_{1}-z\prime_{2}\right)}^{2}}$$
